# Built environment factors and their impacts on outdoor walking activity among people living with dementia: a spatial analysis approach

**DOI:** 10.3389/fpubh.2025.1576548

**Published:** 2025-06-09

**Authors:** Mohammadjavad Nouri, Habib Chaudhury

**Affiliations:** Department of Gerontology, Simon Fraser University, Vancouver, BC, Canada

**Keywords:** neighborhood environment, dementia-friendly community, urban planning and design, GIS, GPS

## Abstract

**Introduction:**

As the population of people living with dementia in Canada continues to grow, understanding the built environment’s role in facilitating outdoor activity is increasingly critical. While prior qualitative and quantitative research has established the benefits of outdoor walking for the physical, mental, and social well-being of people living with dementia, empirical spatial analysis of built environment factors influencing their walking behavior remains limited.

**Methods:**

This study serves as a proof of concept, demonstrating the feasibility of applying spatial analysis to assess the impact of built environment variables on outdoor walking among people living with dementia. Using data from 25 participants in Metro Vancouver, this study integrates Geographic Positioning System (GPS) and Geographic Information System (GIS) tracking with exploratory factor analysis (EFA) and multiple linear regression (MLR) to examine the relationship between built-environment characteristics and walking distances.

**Results:**

Despite the small sample size, statistical analyses met standard validity criteria, identifying three key factors influencing walking distance: (1) Macro environment—accessibility to public transportation and street network characteristics (*p* = 0.007, 439.6 m increase), (2) Micro environment—pedestrian-oriented design (*p* = 0.065, 286.5 m increase), and (3) General characteristics—mixed land use and sidewalk suitability (*p* = 0.015, 388.5 m increase).

**Discussion:**

These findings provide preliminary evidence of the built environment’s role in shaping mobility for people living with dementia, offering valuable insights for public health policy makers, urban planners and designers, and transportation professionals in designing dementia-friendly neighborhoods. By integrating spatial analysis with environmental design principles, this study contributes to the development of inclusive and accessible urban environments for people living with dementia.

## 1 Introduction

Dementia prevalence in Canada is projected to rise significantly, with cases expected to increase from 597,300 in 2020 to nearly 1.7 million by 2050 (1). Data from the Alzheimer Society of Canada (2022) highlight a growing public health challenge, particularly in British Columbia, where the number of people living with dementia is projected to rise from 77,700 in 2020 to 247,300 by 2050—an estimated total increase of 218%. While region-specific data for Metro Vancouver remain limited, its status as the most densely populated area in the province—home to 2.65 million of British Columbia’s 5 million residents across 21 cities (2)—suggests a significant proportion of people living with dementia reside in this metropolitan region.

With 69% of people living with dementia under 80 and 58% of those over 80 residing in community settings rather than long-term care facilities, the neighborhood environment plays a crucial role in supporting their well-being (1). As the population of people living with dementia grows, dementia-inclusive urban planning and policy interventions are essential to fostering accessible, safe, and supportive communities that promote mobility, social engagement, and independence of people living with dementia.

A key policy approach to addressing this need is the Dementia-Friendly Communities (DFCs) framework, which has evolved over the past decades to provide a structured approach for assessing and monitoring various dimensions of the neighborhood environment that influence the outdoor activity and mobility of people living with dementia. It aims to enhance physical and mental well-being, independence, and dignity by fostering accessible, inclusive, and supportive spaces that promote meaningful engagement with the built environment and active participation in community services and programs (3–5).

A dementia-friendly neighborhood environment plays a crucial role in encouraging outdoor activities, such as neighborhood walks, for people living with dementia. By promoting mobility, facilitating access to essential community resources, and fostering engagement with their surroundings, such environments help counteract the “shrinking world” that undermines people living with dementia independence (6, 7). This shrinking world often results from declining abilities, reduced confidence, and structural barriers, including unsafe or unsupportive neighborhood design, social stigma, and mental health-related fears (8–11). Additionally, fear of exposure, getting lost, spatial disorientation, and cognitive decline affecting spatial decisions further restrict mobility, often leading to home confinement (8, 12, 13).

In the existing literature on the impact of neighborhood environments on the outdoor activities of people living with dementia, most scholars have focused on either the social or built environment, with some exploring their interaction through a relational lens. Scholars examining the social environment emphasize the neighborhood’s role in shaping identity, inclusion, and social support for people living with dementia (6, 7, 9, 11). In contrast, research on the built environment adopts an environmental perspective, investigating how urban design, infrastructure, and accessibility features influence neighborhood activity, ultimately affecting the health and well-being of people living with dementia (14–17). Additionally, scholars employing a relational approach to the neighborhood environment highlight coping strategies that enable people living with dementia to navigate and sustain social connections within the community, reinforcing their sense of belonging and inclusion (7, 10, 18–20).

Most research on dementia-friendly neighborhoods has been developed using qualitative methodologies, with relatively few quantitative studies (21). While qualitative research has provided deepened insights into how neighborhood environments influence the outdoor activities of people living with dementia, there remains a need for more robust quantitative methodologies to generate structured and generalizable evidence supporting the role of dementia-friendly neighborhood indicators. To address this gap, recent studies in health and biomedical engineering have begun to focus on quantifying the life space of people living with dementia within neighborhoods by utilizing GPS technology (22, 23). This emerging approach offers a data-driven perspective, enabling researchers to analyze mobility patterns and better understand the spatial behavior of people living with dementia in relation to their environment.

While GPS technology provides valuable insights into outdoor activity patterns among people living with dementia, a comprehensive analysis requires integrating both quantitative and spatial dimensions to fully capture the influence of neighborhood environments on outdoor mobility. Therefore, this study seeks to advance the application of GPS data by enhancing its robustness through the integration of GIS and statistical modeling to analyze how built environment variables shape walking behavior in dementia-friendly neighborhoods.

Although quantitative spatial analysis can be applied to examine the social environment’s impact on outdoor activity, this study focuses specifically on the built environment, as quantitative indicators for assessing the built environment have been more extensively developed. In contrast, research on the social environment has been predominantly qualitative, highlighting the need for further studies to establish quantitative measures in this domain. Aligned with existing GPS-based research predicting outdoor activity patterns for people living with dementia and studies exploring the built environment’s role in shaping their walking experiences, this study aims to address the following research question:

- What neighborhood built environment factors influence the walking activity of people living with dementia within the Metro Vancouver context?
- How do built environment factors predict the overall Regular Walking Route (RWR) distance for people living with dementia?

In our examination of built environment variables influencing the walking experiences of people living with dementia in their neighborhoods, we conducted a review of recent scoping reviews (21, 24). Additionally, we considered other recent studies to extract a set of built environment variables affecting outdoor walking activity of people living with dementia (10, 22, 23, 25).

Previous research has not extensively delved into the quantitative spatial elucidation of walking activity among people living with dementia. For example, using a qualitative methodology, Biglieri and Dean (18) categorized these variables into three primary groups: “land use and transportation,” “urban design,” and “wayfinding.” In our study, we specifically concentrated on built environment variables related to the “land use and transportation” and “urban design” categories. However, we excluded the “wayfinding” category due to limitations in data production and collection for this aspect.

Recent GPS studies investigating outdoor mobility in people living with dementia have employed diverse indicators (22, 23). In contrast, our study specifically focuses on utilizing GPS data to measure outdoor walking activity in the neighborhoods of people living with dementia. We collected data on participants’ regular destinations, home location, and GPS tracks of their RWR to and from these destinations. The inclusion of GPS tracks is vital as it establishes a connection between GPS data and built environment variables.

While we have identified a set of variables to examine how built environment factors collectively predict the overall RWR distance for people living with dementia, it is important to emphasize that this study takes an exploratory approach. Unlike previous research, where variables were categorized into predefined aspects such as land use, transportation, and urban design, our methodology proposes a structured framework of built environment factors tailored to the Metro Vancouver context and evaluates how this structure predicts RWR distance. This structured framework could be applied in future studies with larger participant samples to assess its external validity and further refine its applicability in dementia-friendly neighborhood research.

## 2 Materials and methods

**Figure 1:**
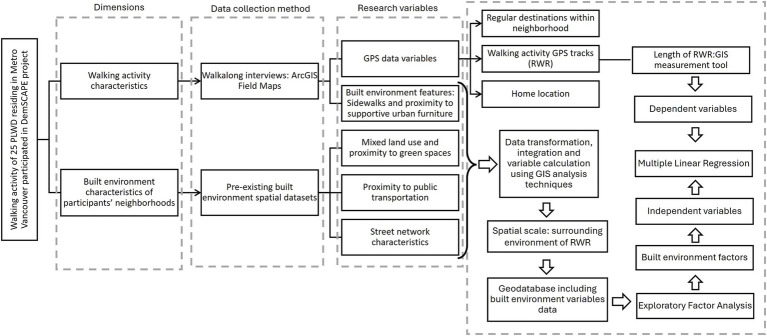
Research process flowchart illustrating the integration of the study’s two main dimensions: walking activity characteristics (length of RWR) and built environment characteristics of participants’ RWRs. The flowchart outlines the research variables, data collection methods, spatial analyses, and statistical modeling techniques, including EFA and MLR. People living with dementia, People Living with Dementia; DemSCAPE, Dementia-Inclusive Streets and Community Access, Participation, and Engagement; GIS, Geographic Information System; RWR, Regular Walking Route; EFA, Exploratory Factor Analysis; MLR, Multiple Linear Regression.

Figure 1 illustrates the research process for identifying built environment factors that influence outdoor activities among people living with dementia and their contribution to RWR length. The framework integrates the initial categories of independent built environment variables derived from literature review, the study’s dependent variable, and the tailored data collection methods used. It also details the selection of relevant research variables and the application of spatial analysis, EFA, and MLR to determine which built environment factors contribute to RWR and to what extent.

This systematically designed methodology aligns with the study’s objectives, offering a comprehensive and multidimensional perspective on people living with dementia’s walking behavior. Each step depicted in Figure 1 is further elaborated upon in the following sections for clarity and depth.

### 2.1 Participants and study area

This study is part of the broader Dementia-Inclusive Streets and Community Access, Participation, and Engagement (DemSCAPE) project, which aims to improve outdoor mobility, social participation, and community engagement for people living with dementia. The primary objective was to identify key neighborhood destinations and built environment features that support people living with dementia in their everyday outdoor activities (26).

To recruit participants, a multi-faceted approach was employed, including flyer and poster distribution, social media outreach, collaboration with local community organizations, referrals from a memory clinic, and invitations to individuals who had participated in previous research projects.

The study sample comprised 32 community-dwelling individuals diagnosed with mild to moderate dementia or mild cognitive impairment, living in urban and suburban areas of Metro Vancouver (26 participants) and Prince George (six participants), British Columbia. Inclusion criteria required participants to self-report a diagnosis of dementia or cognitive impairment, reside in the community, be independently mobile (with or without assistive devices), engage in outdoor walking regularly or occasionally, and be proficient in English.

Ethical approval for the study was granted by the Research Ethics Office (protocol number H21–02461). Two consent forms were designed to accommodate participants’ needs: one for people living with dementia and their care partners (CPs) as dyads, and another with an additional signature line for cases where a CP provided support or acted as a proxy. This flexible consent process was intended to uphold the autonomy and personhood of people living with dementia by allowing dyads to determine how consent was provided. All participants were informed about the study using an ethics-approved script and gave informed consent before taking part.

Data collection was conducted with 25 participants from various cities and districts within Metro Vancouver. Among them, 11 participants completed the walk-along interviews independently, while 14 were accompanied by their CPs. In terms of living arrangements, 17 participants resided with others, while the remaining eight lived alone.

### 2.2 Walking activity characteristics and GPS data

**Figure 2:**
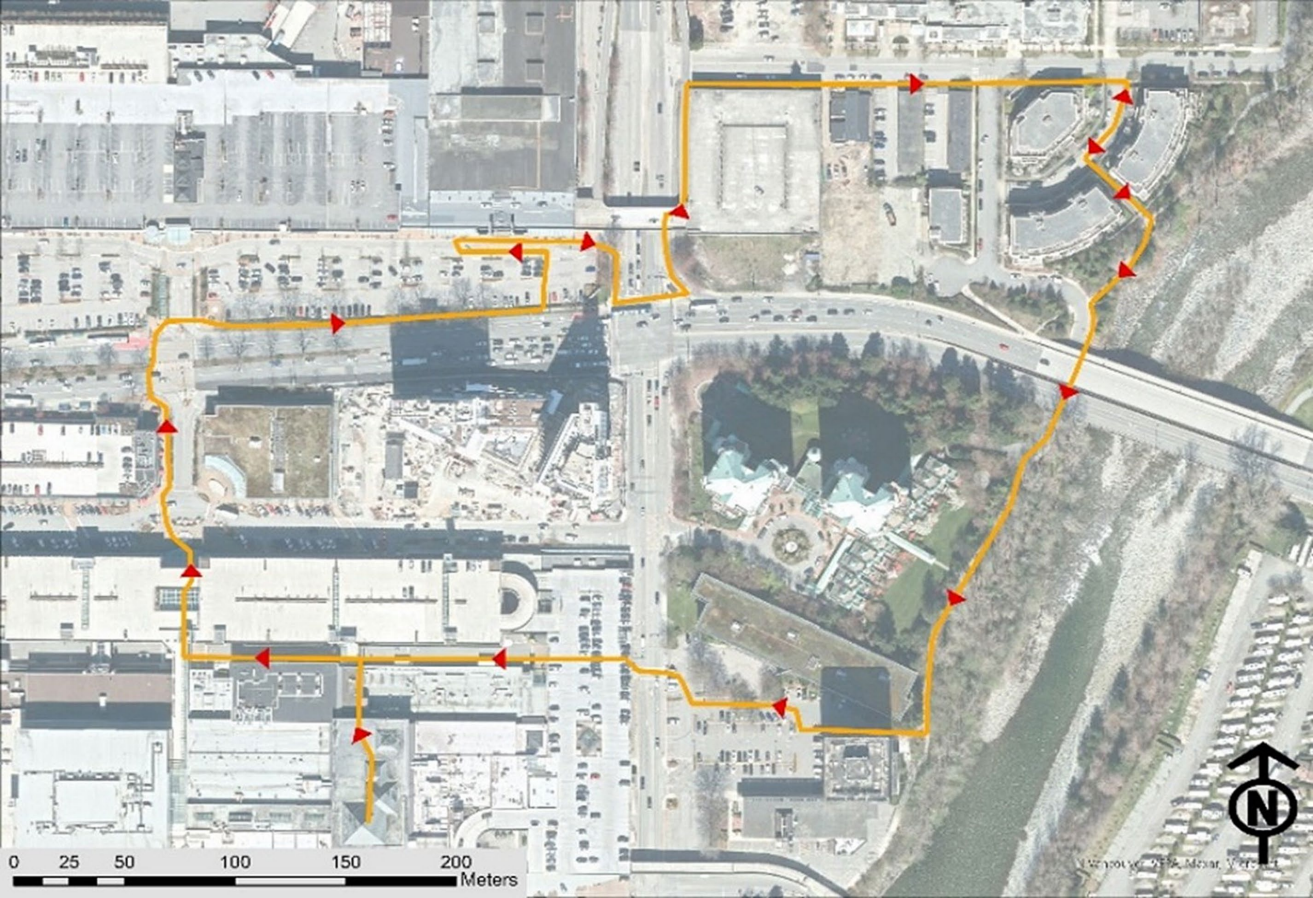
An illustration of a regular walking route (RWR) undertaken by a participant.

This study utilized GPS data obtained from 25 walk-along interviews with people living with dementia, conducted between June and December 2022 as part of the DemSCAPE project. Each participant was interviewed once while walking along their RWR to a familiar neighborhood destination (Figure 2).

To document home locations, RWRs, and frequent destinations, an online geodatabase was created for each participant using the ArcGIS Online platform. These geodatabases were then integrated into the ArcGIS Field Maps application on an Apple iPad (9th generation) for data collection (26). During the walk-along interviews, research assistants accompanied participants along their RWRs, recording GPS tracks and capturing built environment attributes relevant to the study’s research variables using ArcGIS Field Maps. To ensure data accuracy, the collected GPS tracks and environmental feature recordings were exported to ArcGIS Pro 3.2.0 for further processing. Research assistants who conducted the walk-along interviews collaborated with the team’s GIS analyst, cross-referencing the data with OpenStreetMap and Google Satellite imagery to verify and refine the RWR GPS tracks and associated environmental features.

### 2.3 Variables, data sets, and method of calculation

#### 2.3.1 Dependent variable and spatial scale of analysis

As this study aims to examine how built environment factors contribute to the length of the RWR, we have identified one primary dependent variable, defined as follows:

- Length of RWR: The total distance (in meters) traveled from the participant’s home to their regular destination and back. This distance was calculated using the length measurement tool in ArcGIS Pro 3.2.0, which measures the GPS track recorded for each RWR.

The spatial scale of analysis for calculating independent built environment variables is the surrounding environment of the RWR. The definition of this surrounding environment varies depending on the nature of each variable, which will be discussed in detail in the following section.

All independent built environment variables were calculated at this spatial scale and analyzed using EFA to identify key built environment components. These components were then tested using MLR to assess their predictive power for RWR distance.

#### 2.3.2 Independent built environment variable

##### 2.3.2.1 Land use mix and proximity to green spaces

To evaluate land use mix within the surrounding environment of the RWR, this study applied the entropy index formula recommended by Bordoloi et al. (27). to quantify land use mix level in parcels adjacent to the RWR. Additionally, the average Euclidean distance from the RWR to green spaces was computed. These spatial metrics were processed and integrated into each participant’s RWR layer using ArcGIS Pro 3.2.0 for further analysis.

##### 2.3.2.2 Proximity to public transportation

To assess proximity to public transportation stops and routes within both the 20-min walkshed and the surrounding environment of the RWR, multiple spatial variables were calculated using the Euclidean distance method in ArcGIS Pro 3.2.0. Key measures included:

- Average distance to bus stops: the average distance of RWR to bus stops.
- Average distance between bus stops, considering those within a 20-meter buffer of the RWR.
- Number of bus stops within the 20-meter buffer of the RWR.
- Number of bus routes to different destinations within the 20-meter buffer of the RWR.

Data for public transportation stops and routes were sourced from the GTFS dataset provided by TransLink, the region’s primary public transit provider.

#### 2.3.3 Street network characteristics and proximity to supportive urban furniture

To evaluate street network characteristics and proximity to supportive urban furniture, multiple spatial variables were calculated using various platforms, methods, and datasets. Key measures included:

- Average slope (percentage) of RWR, determined using elevation profiles from Google Earth and assigned to each participant’s RWR GIS layer.
- Percentage of overlap between RWR and different street function levels including.
  - Residential,
  - Secondary,
  - and non-motorized streets such as pedestrianized paths, cycleways, and community paths, calculated using OpenStreetMap data in QGIS Desktop 3.2.8.
- Percentage of overlap between sidewalks and RWR, based on field data collected during walk-along interviews using ArcGIS Field Maps.
- Percentage of overlap between bike lanes and the RWR, calculated using bike lane dataset sourced from the Metro Vancouver Open Data Portal (2023).
- Number of benches along the RWR, based on field data collected during walk-along interviews using ArcGIS Field Maps.
- Total number of four or more-way intersections along the RWR.

### 2.4 Statistical analysis

To address the two main research questions, we employed two statistical modeling techniques, each corresponding to one question. First, to identify the built environment factors influencing neighborhood walking activity among people living with dementia in Metro Vancouver, we conducted EFA using independent variables as inputs. Given the small sample size (25 participants), we carefully assessed statistical assumptions before proceeding. Normality was evaluated through descriptive statistics and graphical methods such as histograms and Q-Q plots (28, 29). Additionally, we applied the Kaiser-Meyer-Olkin (KMO) Measure of Sampling Adequacy to assess the suitability of the data for factor analysis and conducted Bartlett’s Test of Sphericity to confirm the presence of sufficient correlations among variables (30). These tests were performed in SPSS version 27, ensuring that the data met the necessary conditions for factor extraction.

Recognizing the limitations of the small sample size, this study serves as a proof of concept, establishing a methodological foundation for future research with larger datasets. EFA was chosen as a dimension reduction technique to manage the high number of built environment variables relative to the limited sample size. Since an excessive number of predictors can compromise model stability in regression analysis, EFA was used to extract latent factors representing key built environment characteristics shaping people living with dementia’s walking experiences. Principal Components Analysis (PCA) was employed as the factor extraction method, with varimax rotation applied to construct a simple, distinct structure of built environment factors.

Second, the built environment factors identified through EFA were subsequently used as predictor variables in a MLR model to estimate the length of the RWR. Given that varimax rotation produces uncorrelated factors, multicollinearity was not assessed. The final MLR model was tested for overall model fit (Adjusted R^2^ and ANOVA F-test) to validate its predictive capacity (31).

By integrating EFA and MLR within an SPSS-based analytical workflow, this study provides an empirical framework for examining the built environment’s role in influencing people living with dementia’s mobility. Future research can expand on this approach with larger samples to strengthen the external validity of these findings.

## 3 Results

GPS tracking data revealed that participants walked an average distance of 1 km from home to their regular neighborhood destination, with distances ranging from 239 to 1,908 meters. Nearly half walked less than 1 km, while the others covered 1 to 2 km. The analysis further indicated an average RWR length of 1,963 meters, ranging from 470 to 4,165 meters. Approximately 60% of RWRs were shorter than 2 km, while 40% exceeded this distance.

### 3.1 Question 1: built environment factors and walking activity

EFA depends on the assumption of a normal distribution in the data. To confirm this assumption, skewness and kurtosis coefficients were computed, and histograms and Q-Q plots were inspected for all 14 variables. The results revealed that all 14 variables followed a normal distribution.

The KMO and Bartlett’s Test of Sphericity were computed to evaluate the appropriateness of conducting EFA. The KMO value of 0.527 and the significant result for Bartlett’s Test of Sphericity (Sig. = 0.0001) indicated that the dataset was deemed mediocre for EFA, potentially due to the limited sample size (31).

To ascertain the number of factors influencing participants’ RWR distance, we initially applied the Kaiser criterion. Extracting factors with eigenvalues equal to or exceeding 1.0 yielded four factors, collectively elucidating about 72% of the data variance. Employing a scree plot indicated that three factors sufficed to explain the model’s objective (64% of data variance). Comparing the outcomes of the three and four-factor structures and aligning them with pertinent literature on the outdoor walking experience of individuals with dementia, we selected the three-factor structure based on scree plot insights. Subsequently, Varimax rotation was employed to simplify the structure and unveil the factors influencing the outdoor walking experience of individuals with dementia in Metro Vancouver.

**Figure 3:**
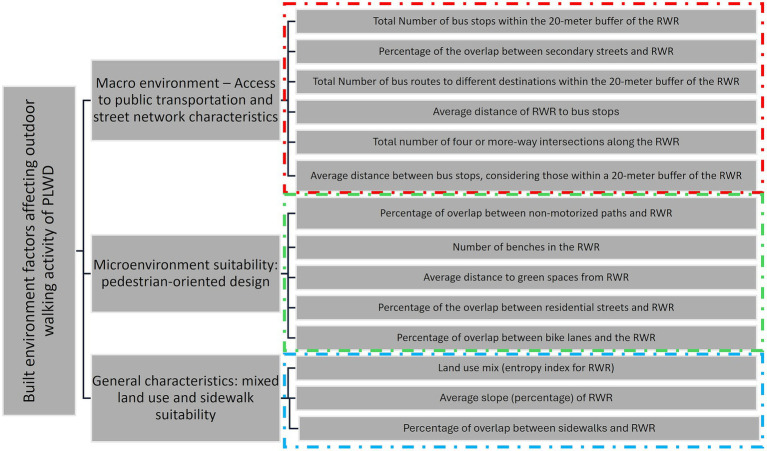
The three-factor structure of the built environment variables explaining the outdoor walking experience of people living with dementia (people living with dementia).

The configuration of the three extracted factors is depicted in Figure 3. These factors are labeled as (1) Macroenvironment: Access to public transportation and street network characteristics, (2) Microenvironment: Pedestrian-oriented design, and (3) General characteristics: Mixed land use and sidewalk suitability, explaining 28, 20, and 15% of the variation in the data pertaining to the outdoor walking experience of people living with dementia, respectfully.

#### 3.1.1 Macroenvironment: access to public transportation and street network characteristics

This factor predominantly addresses the access to public transportation, specifically focusing on bus stops and bus lines. It also encompasses street network characteristics related to secondary streets and the frequency of 4 or more-way intersections. Secondary streets, designed for lower traffic volumes compared to primary streets or trunk ways, traverse residential areas, and often feature mixed land use, extensive pedestrian infrastructure, safety measures such as separated bike lanes and sidewalks, as well as adequate traffic signals and signs. The high correlation among these variables and their amalgamation into a factor underscores the significance of public transportation services primarily being provided on secondary streets. This type of street shapes the macro-level environment for residents within a neighborhood. Four or more-way intersections in these streets typically provide pedestrians with a good level of safety and comfort to traverse the intersections. This suggests that built environment features are not only crucial in the immediate home surroundings and the micro-surrounding area of destinations but also play a role for participants interested in walking longer distances, where the macro-surrounding area becomes influential.

#### 3.1.2 Microenvironment: pedestrian-oriented design

This factor characterizes the micro-surrounding environment, typically near the homes of people living with dementia and the buffer areas surrounding their destinations. The variables within this factor denote a pedestrian-oriented design of the micro-environment, encompassing the local access street network. This network includes paths dedicated to non-motorized functions such as pedestrianized paths, paths within buildings (e.g., community centers, shopping centers, university campuses), and open spaces like beaches, parks, trails, and green spaces. Residential streets are also part of this network, serving various functions in the micro-surrounding environment of people living with dementia, such as accessing local destinations or acting as hubs for transferring to secondary streets for longer-distance destinations (transfer to macro environment). The presence of benches in these environments is crucial, whether people living with dementia is heading for a long-distance or a nearby destination, as they often use residential streets for their outdoor walking. The inclusion of separated bike lanes is vital in this micro-surrounding built environment to ensure convenience, especially given the potential interaction with cyclists. All these variables exhibit correlation relations within a factor constructed to represent the pedestrian environment at the micro-level, distinguishing it from variables highly correlated with those included in the first factor.

#### 3.1.3 General characteristics: mixed land use and sidewalk suitability

While the two previous factors were associated with built environment variables related to the macro and micro-surrounding environments, this factor pertains to more general characteristics of the surrounding built environment. It encompasses variables such as the slope of the RWR and the presence of sidewalks within RWR. Both variables highlight the ease of walking in a built environment. However, their correlation with other variables, land use mix, suggests that merely providing sidewalks and minimizing the slope of RWR is not sufficient. There should also be a sense of diversity in activities surrounding the sidewalks and RWR.

### 3.2 Question 2: predicting RWR length using built environment length

This section reveals the outcomes of our MLR analysis, aiming to predict the RWR distance for people living with dementia in their neighborhoods. Leveraging insights from the EFA, three essential factors were incorporated: “Macroenvironment – Access to public transportation and street network characteristics,” “Microenvironment suitability: pedestrian-oriented design” and “General characteristics: mixed land use and sidewalk suitability.”

The regression model demonstrated a robust fit, elucidating 48.5% of the variance in RWR length. The Adjusted R Square, factoring in predictor numbers, stood at 41.1%. With a Std. Error of the Estimate of 719.838, representing the average deviation between observed and predicted values, the model’s precision is apparent. ANOVA further corroborated the model’s significance, with the F-statistic (6.581, *p* = 0.003) affirming the collective impact of predictors on journey distance.

Key coefficients in the analysis yield valuable insights. The intercept (Constant) at 1963.240 establishes the baseline RWR length. The “Macro environment – Access to public transportation and street network characteristics” exhibits a substantial positive effect (*p* = 0.007) at a 95% confidence level, indicating that a one-unit increase corresponds to a noteworthy 439.643-unit rise in RWR length, with a standardized beta coefficient of 0.469. Similarly, “Micro-environment: Pedestrian-oriented design” demonstrates a moderately positive impact (*p* = 0.065) at a 90% confidence level, suggesting that a one-unit increase in this factor results in a 286.465-unit increase in RWR length, with a standardized beta coefficient of 0.305. Additionally, “General characteristics: mixed land use and sidewalk suitability” shows a significant positive influence (*p* = 0.015) at a 95% confidence level, with a coefficient of 388.510 and a standardized beta coefficient of 0.414. This implies that a one-unit increase in “General characteristics: land use mix and sidewalk suitability” corresponds to a substantial 388.510-unit increase in journey distance.

## 4 Discussion

Facilitating outdoor walking activities for people living with dementia is a fundamental objective of developing DFCs. However, this relationship is multidimensional, influenced by several factors. Researchers from various disciplinary backgrounds have explored it from their respective perspectives. Yet, examining this relationship through the lens of spatial analysis, adds a spatial dimension that aids in identifying which factors are most influential. This approach contributes to the development of effective strategies for creating dementia-friendly outdoor environments.

This study aimed to investigate the predictive capacity of built environment variables on the walking activity length of people living with dementia in their neighborhoods. Despite our study’s limited sample size of 25 participants, both EFA and MLR model support the validity of our findings.

The results of the EFA align with the built environment categories proposed by Biglieri and Dean (18). However, our exploratory data analysis approach revealed that the variables within the suggested categories by them may be subject to contextual differences. For example, in the context of metro Vancouver, access to public transportation and street network characteristics tend to be more correlated, leading to the construction of a factor, while the land use mix pattern is more correlated with the suitability of sidewalks and lower slope percentage of the RWR, forming a new factor. Nevertheless, the factors identified in our study and their suggested categories exhibit overlaps, reinforcing and validating each other.

The study reveals statistically significant positive impacts for various factors influencing the walking activities of people living with dementia in their neighborhood. Despite the complexity introduced by various variables within each factor, the model results emphasize that enhancing aspects related to public transportation, intersections, pedestrian-oriented design, and general characteristics like land use and sidewalk suitability can statistically encourage people living with dementia to engage in more extended walking activities within their neighborhood.

**Figure 4:**
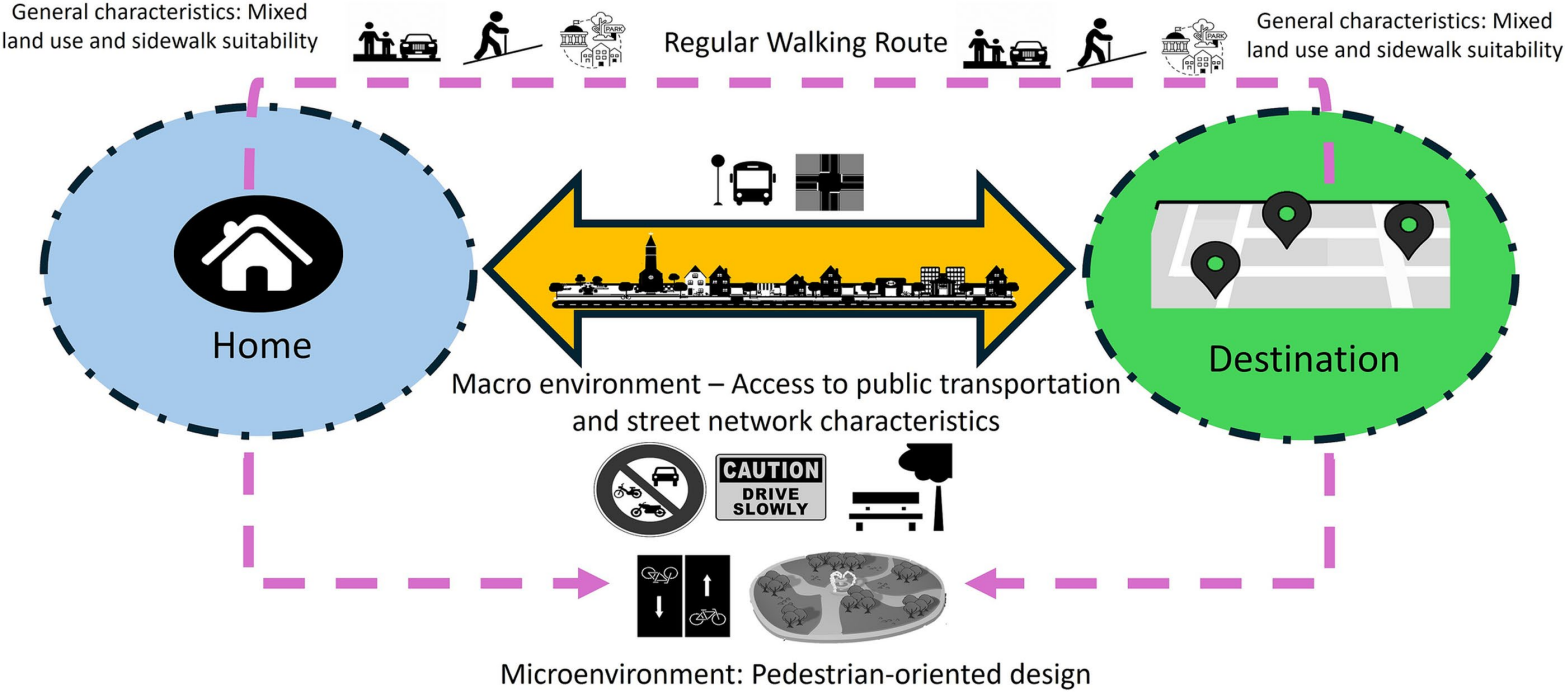
A scheme of built environment factors and their related variables affecting outdoor walking activity of people living with dementia (people living with dementia) based on the model results for Metro Vancouver.

The results of the EFA informed the development of a framework that identifies which built environment factors and variables influence outdoor walking activity among people living with dementia and at what spatial scale (Figure 4). The findings suggest that analyzing people living with dementia’s outdoor walking activity should consider two levels of built environment characteristics across distinct spatial scales. The first level pertains to the micro surrounding environment, primarily associated with the nearest destinations and the proximity of people living with dementia homes. The second level involves the macro environment, highly connected more distant destinations. This suggests the need for a two-tiered approach in urban design policies when aiming to enhance the neighborhood environment for people living with dementia. Additionally, two overarching considerations related to sidewalks and mixed land-use should be addressed at both scales. Subsequent sections will delve into a discussion of our study results in relation to previous research findings.

### 4.1 Macro environment accessibility: public transportation and street network

This finding is consistent with prior studies emphasizing the importance of accessible public transportation systems in fostering age-friendly and dementia-friendly communities. These systems, especially when equipped with easily identifiable signs, seating, and shelters, can also serve as rest areas for people living with dementia (14, 18, 32–34).

The factor loading of 0.930 for the “Number of bus stops within a 20 m buffer of RWR” indicates a highly positive correlation between this indicator and the macro environment accessibility factor. This suggests that a higher number of bus stops within a 20-min buffer of RWR correlates with an increased level of accessibility in the macro environment. While some scholars posit that a higher number of bus stops in an area is positively correlated with sedentary time (57), our study suggests that, in the context of macro environment accessibility, this variable contributes to longer distances covered in walking activities.

Additionally, the “Number of bus routes along with the RWR,” with a factor loading of 0.744, demonstrates a positive correlation with the accessibility of the macro environment. This variable provides more transportation options to various destinations, thereby encouraging longer walking activities by contributing to the overall accessibility of the macro environment.

Our findings reveal a negative correlation between the “Average distance to bus stops from the RWR” and the accessibility of the macro environment, indicated by a factor loading of 0.737. This implies that a shorter average distance to bus stops along the RWR is associated with a more accessible macro environment, contributing to longer RWR. Interestingly, the accessibility of the macro environment also increases with an increase in the “Average distance between bus stops located within a 20 m buffer of the RWR,” as evidenced by the factor loading of “0.484.” However, it is noteworthy that, based on the coefficients, the average distance to bus stops from RWR appears to be more strongly correlated with the factor than the average distance between bus stops.

Our study suggests that a higher “Percentage of the overlap between secondary streets and RWR” positively influences the accessibility of the macro environment, as evidenced by the loading factor of 0.880. This, in turn, leads to longer RWR. This result aligns with previous research emphasizing the significance of street hierarchy in route selection among people living with dementia. People living with dementia typically avoid noisy, high-traffic, fast-moving arterial roads, including primary and trunk roads. Instead, they prefer quieter residential or mixed-use streets that provide a buffer from heavy vehicular and bike lane traffic, prioritizing their safety (6, 16, 17, 19, 33–39).

Furthermore, secondary streets should exhibit distinct built forms, especially those designed as pedestrian-oriented streets, to aid people living with dementia in wayfinding (14). For participants aiming for longer durations and greater distances, secondary streets offer a more accessible macro environment with wider sidewalks, fewer obstructions, and accessible crossing points (17).

Consistent with previous research indicating a link between low intersection density and instances of jaywalking, and higher intersection density promoting walkability (10), our study reveals that, for people living with dementia, a higher “Number of 4 or more-way intersections in the RWR” is correlated with a heightened level of accessibility in macro environments. This is evidenced by a factor loading of 0.656, contributing to extended walking activity for people living with dementia. Among our participants, those interested in longer walking distances typically opt for secondary streets to reach their destinations. Secondary streets, characterized by a higher number of intersections, are equipped with crosswalks featuring auditory and visual signals, thereby enhancing the sense of security for older adults specially people living with dementia (40).

### 4.2 Microenvironment suitability: pedestrian-oriented design

The factor loading of 0.838 for the “Percentage of overlap between non-motorized paths and the RWR” indicates a highly positive correlation between the presence of non-motorized routes and the pedestrian-oriented design of the micro-scale built environment. These pathways, dedicated to non-motorized functions such as pedestrianized paths within buildings (e.g., community centers, shopping centers, university campuses), beaches, parks, trails, and green spaces, offer crucial support for people living with dementia in terms of accessibility.

These paths are inherently barrier-free and accessible, addressing key issues that limit walking distances for individuals. Barriers, such as busy roads, railway lines, bodies of water, steep slopes, fences, and private property, negatively impact walking distances and hinder the creation of dementia-friendly communities (34). Moreover, these paths are designed with features that enhance safety and usability. Importantly, they are free from obstacles like bus queues, cars parked over sidewalks, and poorly placed street furniture, all of which can cause anxiety for people living with dementia (41, 42).

Additionally, these paths minimize exposure to noise, a crucial factor in environmental quality. Positive noises, like children playing or navigational sounds, and negative noises, such as traffic and screaming, can significantly impact individuals with reduced cognitive function, including dementia. People living with dementia may actively choose these routes to avoid disturbing sounds. Non-street environments, including trails, footpaths, and green spaces, are preferred by older adults, promoting increased walking and physical activity (40). However, it is essential to ensure that these paths are flat, even, well-kept, located in low-crime areas, and include marked level changes and handrails where necessary to promote a steady gait, particularly for people living with dementia (14).

The “number of benches in the RWR” is highly positively correlated with the suitability of the microenvironment regarding pedestrian-oriented design, as demonstrated by the factor loading of 0.706. This indicates that an increased number of benches in the RWR corresponds to an increase in RWR distance. Our study aligns with prior research emphasizing the benefits of street furniture, specifically benches, for older adults with dementia. Street furniture contributes to establishing a human-scale environment, enhancing legibility, and providing opportunities for rest (43). Earlier studies have highlighted how insufficient seating, and a lack of restrooms can restrict mobility for older adults, particularly those with dementia (36, 55, 56).

The factor loading of −0.686 for “Average distance to green spaces from the RWR” indicates a negative correlation with the pedestrian-oriented design of the micro environment. This implies that a shorter distance from green spaces corresponds to a more suitable pedestrian-oriented environment, contributing to longer walking activity for people living with dementia. Our findings align with previous research, which suggests that proximity to green spaces positively influences the walking activity of older adults with dementia. This proximity provides leisure opportunities, a social environment, enhances neighborhood satisfaction, and offers locations for planned physical activities and programs that boost self-esteem (7, 9, 18, 41, 44).

The factor loading of −0.599 for the “Percentage of the overlap between residential streets and the RWR” indicates a negative correlation with the pedestrian-oriented design of the micro environment. This implies that, for longer distances of walking activity, people living with dementia tend to use the residential environment as a pathway to access other levels of functional streets, including secondary streets as evidenced under the first factor, rather than exclusively choosing residential streets. It is important to note that this negative correlation does not imply that their walking activity is less overlapped with residential streets. Some participants may solely use residential streets, particularly if they choose to walk shorter distances.

The “Percentage of bike-line overlap with the RWR” is positively correlated with the pedestrian-oriented design of the microenvironment evidenced by factor loading of 0.511. This suggests that a higher presence of pedestrian infrastructure, such as separator lanes for cyclists, contributes to the increased suitability of the microenvironment, thereby enhancing the potential for longer walking activity among people living with dementia. These findings align with previous research that underscores the importance of addressing user conflicts when different types of users share the same space. This involves mitigating obstructions, such as bus queues, and ensuring clear separation between cyclists and pedestrians (42).

### 4.3 General characteristics: mixed land use and sidewalk suitability

The factor loading of 0.636 for “Mixed land use (entropy index for RWR)” indicates a positive correlation with the general characteristics influencing the suitability of both macro and micro environments. An increase in the mixed-use pattern in the surrounding environment of people living with dementia’s walking activity is associated with longer RWR. This mixed-use environment provides more destinations accessible by foot, enhancing distinctiveness and potentially reducing disorientation and the risk of getting lost for people living with dementia. These findings align with previous studies emphasizing the positive effects of access to natural environments, public facilities, community centers, and retail shops on independence, social interaction opportunities, cognitive function, emotional well-being, and walking engagement for people living with dementia (10, 13, 14–16, 25, 45–47).

**Figure 5:**
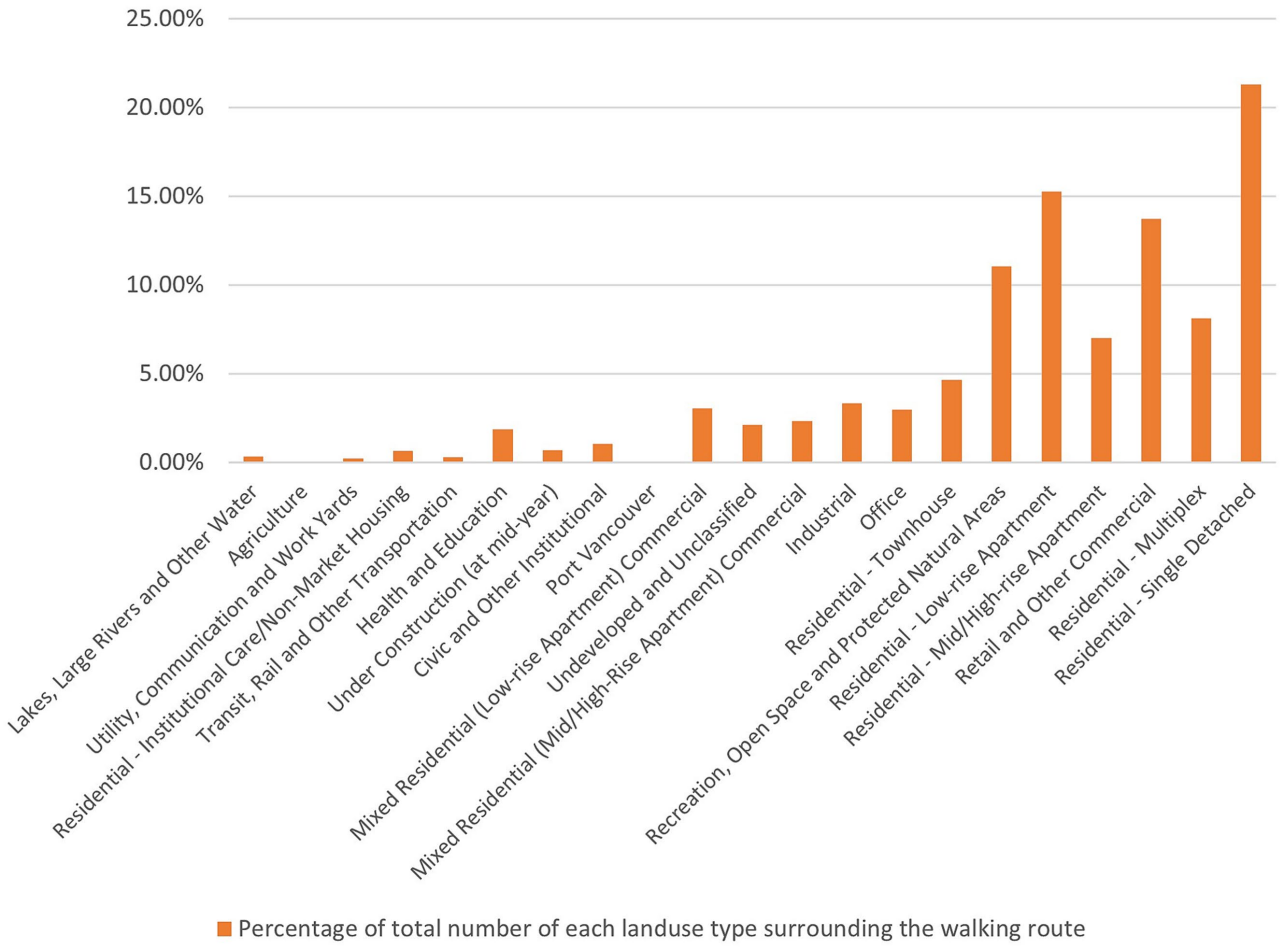
Percentage of total number for each land use type along RWRs.

**Figure 6:**
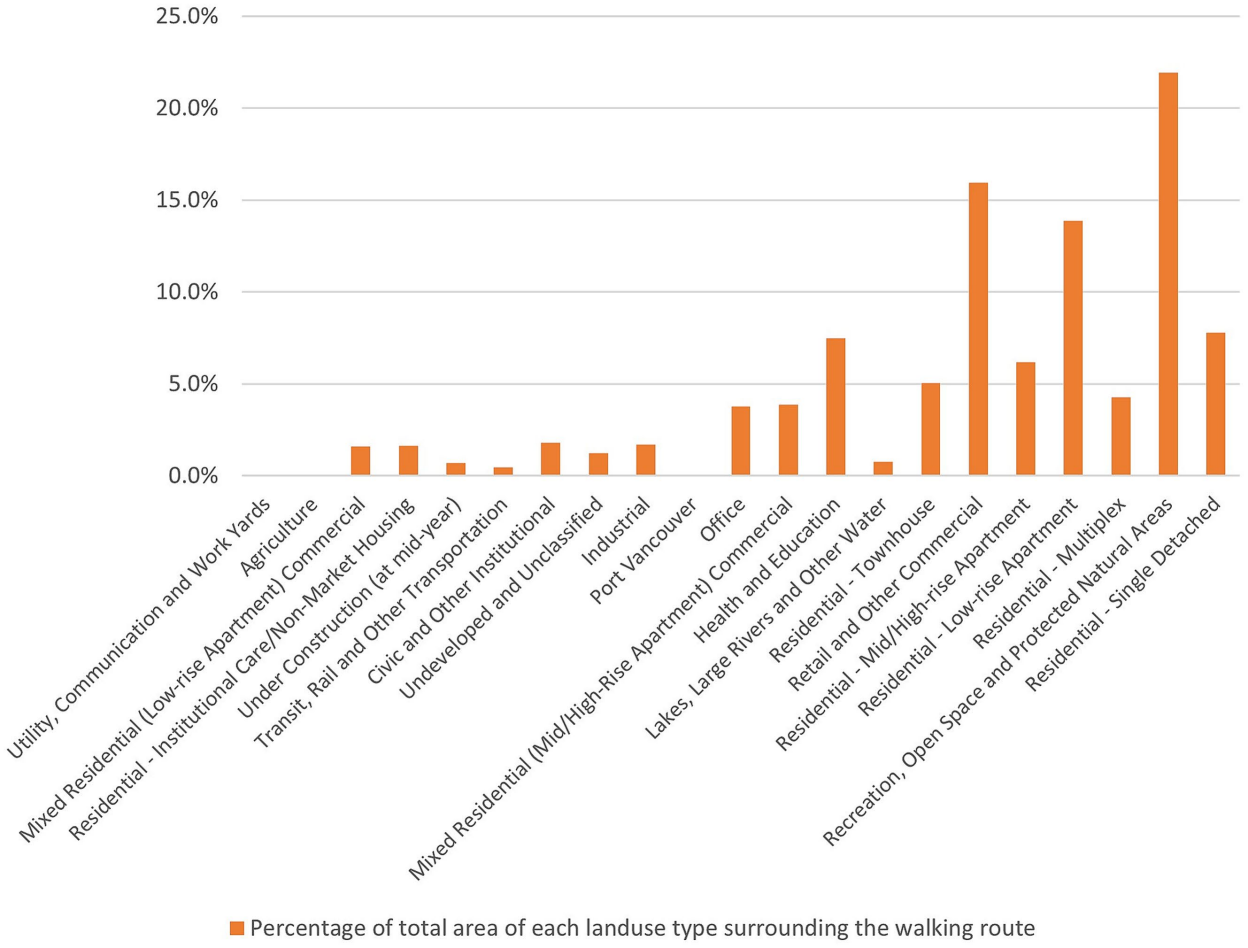
Percentage of total area for each land use type along RWRs.

Participants in our study exhibited a preference for RWRs with diverse land uses, encompassing residential, retail, commercial, recreational, open space, and protected natural areas, along with apartments, parks, natural areas, and community facilities (Figures 5, 6). Facilities for recreation and community engagement, such as community gardens, community centers, third places, religious establishments, and public arts, significantly impact the outdoor walking activity of people living with dementia by providing opportunities for community involvement, social interaction, and participation. These elements serve as landmarks and points of interest for wayfinding, enhance neighborhood satisfaction and a sense of belonging, offer respite from crowded areas, and often include rest stops such as benches and public restrooms. This array of amenities supports the well-being and outdoor activities of people living with dementia (9, 14, 34, 41, 48, 49). Retail and commercial areas significantly influence the outdoor walking activity and mobility of older adults due to their popularity among this demographic, their positive effects on older adults’ social capital, fostering a greater sense of community, and contributing to pedestrian-friendly shopping opportunities (18, 34, 44, 48).

The factor loading of −0.770 for “Average of RWR slope” indicates a negative correlation with the suitability of both macro and micro built environments. A lesser slope in RWR contributes to a higher suitability of the built environment for longer walking activities. Our results align with previous research emphasizing the importance of flat routes in mitigating challenges associated with an unsteady gait, particularly for people living with dementia (50). Such barriers not only limit the time and distance of walking but also hinder the development of dementia-friendly communities by reducing the independence of older adults.

The factor loading of −0.675 for “Percentage of the overlap between sidewalks and RWR” indicates a negative correlation with the suitability of both macro and microenvironments. For longer walking distances, the presence of sidewalks in the RWR might be deficient, leading to a decrease in the suitability of the sidewalk, especially in the macro-built environment. Our findings suggest that as RWR length increases, participants are more likely to encounter sidewalk network disconnections in metro Vancouver, resulting in the unsuitability of sidewalks. While most participants have access to sidewalks in their chosen RWRs, this access diminishes as they walk longer distances. The presence and quality of sidewalks significantly impact walkability, with poor-quality sidewalks (e.g., cracked, or uneven) posing injury risks (49, 51).

## 5 Conclusion

As the number of people living with dementia continues to rise in Canada, the significance of the built environment in the cities and neighborhoods they inhabit becomes increasingly crucial. According to statistics released by the Alzheimer Society of Canada (1), a substantial portion of this population resides in their homes rather than long-term care facilities. This underscores the pressing need for a more profound understanding of the impact of built environment variables on their outdoor activities in the neighborhood. Outdoor walking activity stands out as one of the most vital pursuits, offering people living with dementia an opportunity to avoid social isolation and enhance their mental and physical well-being.

Previous research, both quantitative and qualitative, has delved into various aspects of the outdoor built environment, exploring the intersections of sociology, built environment, and health. However, there has been limited attention given to the potential of leveraging new technologies such as GPS and GIS to investigate the intricate relationships between these environmental factors and the outdoor activities of people living with dementia. Furthermore, to our knowledge, there are no other studies that used EFA and MLR to build a model to predict outdoor walking activity of people living with dementia based on spatial analysis of built environment variables.

Our findings not only align with previous studies that establish the association between built environment variables and the outdoor walking activity of people living with dementia, but they also contribute to understanding how these built environment features can predict the length of their RWR. The study specifically focuses on two levels of built environment variables and one general characteristic of the built environment, namely: “Macro-environment – Access to public transportation and street network characteristics,” “Micro-environment: Pedestrian-oriented design,” and “General characteristics: Mixed land use and sidewalk suitability.”

These three factors and their associated variables offer valuable guidance for policymakers, urban planners and designers, landscape designers, and transportation planners in developing dementia-friendly neighborhood plans. Establishing criteria and indicators for a dementia-friendly plan in both macro and microenvironments can inform spatial decisions in several ways.

Although all three built environment factors contribute significantly to walking distance and offer actionable guidance for spatial planning and neighborhood design, the macroenvironment—particularly connected street networks and access to public transportation—emerged as the strongest and most statistically significant predictor. As such, it should be prioritized as the most urgent intervention target in dementia-friendly neighborhood planning.

In the macro environment, transportation planners play a critical role in ensuring safe intersections, convenient access to public transportation, and the provision of suitable sidewalks with proper slopes. These considerations are particularly important for people living with dementia undertaking longer RWR to reach distant destinations. Urban planners can collaborate with transportation planners to create a mixed-use environment that caters to the diverse needs of people living with dementia, providing essential services within easy reach.

In the microenvironment, urban and landscape designers are instrumental in crafting pedestrian-oriented environments surrounding people living with dementia’s homes and destinations. While our study did not precisely delineate the spatial extent of these microenvironments from participants’ homes and destinations, it suggests that urban designers should begin with the nearest areas of their homes and key destinations. This approach involves incorporating supportive furniture, green spaces, and safety measures to minimize motorized traffic and bicycle intersections, thus creating a conducive environment for people living with dementia.

Future studies could strengthen their methodologies by incorporating spatial analysis toolkits, thematic mapping, and sentiment analysis to gain a more comprehensive understanding of how people living with dementia navigate and interact with their neighborhood environments. The framework proposed in this study could be extended to identify and map specific intervention zones, enabling targeted improvements in dementia-friendly neighborhood planning.

While the current study focused on identifying built environment predictors of walking activity, future research should also explore the development of spatial monitoring and assessment tools that integrate GPS data, environmental mapping, and observational insights. Such tools would support the continuous evaluation of neighborhood accessibility and guide evidence-based decision-making—particularly by municipal planners—aimed at enhancing walkability, safety, and inclusivity for people living with dementia.

Notably, our study lacks consideration for points of interest and landmarks within the built environment, which have been shown in previous research to influence outdoor walking experiences (6, 25, 52–54).

It’s essential to acknowledge the geographical limitation of our study, which focuses solely on the Metro Vancouver region. Consequently, the generalizability of our findings to other regions or countries may be constrained. Furthermore, weather conditions, particularly in the Canadian context, can significantly influence outdoor walking experiences but were not explicitly addressed in our study.

Another limitation relates to individual-level health factors. While all participants were independently mobile, we did not assess or control variability in functional status, such as visual or hearing impairments, or use of mobility aids, which may have affected walking distance and behavior.

Additionally, the role of social support and walking companionship was not explicitly analyzed. Fourteen participants were accompanied by care partners during data collection, which may have influenced route choice, perceived safety, or willingness to walk longer distances. As dyadic dynamics can play a significant role in outdoor mobility for people living with dementia, this factor warrants further investigation in future research.

Lastly, the small sample size of 25 participants restricts the broad applicability of our results and warrants caution when interpreting and extrapolating findings to larger populations.

## Data Availability

The datasets presented in this article are not readily available due to restrictions imposed by the funding agency’s policy. Requests to access the datasets should be directed to Habib Chaudhury, chaudhury@sfu.ca.
